# Subtype I (intrinsic) adenomyosis is an independent risk factor for dienogest-related serious unpredictable bleeding in patients with symptomatic adenomyosis

**DOI:** 10.1038/s41598-019-54096-z

**Published:** 2019-11-27

**Authors:** Sho Matsubara, Ryuji Kawaguchi, Mika Akinishi, Mika Nagayasu, Kana Iwai, Emiko Niiro, Yuki Yamada, Yasuhito Tanase, Hiroshi Kobayashi

**Affiliations:** Department of Obstetrics and Gynecology, Nara Medical University, Nara, Japan

**Keywords:** Drug regulation, Risk factors

## Abstract

We aimed to retrospectively analyze the risk factors of a continuous dienogest (DNG) therapy for serious unpredictable bleeding in patients with symptomatic adenomyosis. This is a retrospective study based on data extracted from medical records of 84 women treated with 2 mg of DNG orally each day between 2008 and 2017. 47 subjects were excluded from the original analyses due to an inadequate subcategorization into subtype I and subtype II and a lack of hemoglobin levels. The influence of various independent variables on serious unpredictable bleeding was assessed. Of the 37 eligible patients who received the continuous DNG therapy, 14 patients experienced serious unpredictable bleeding. Univariate analysis revealed that the serious bleeding group had subtype I adenomyosis (P = 0.027). There was no correlation between age, parity, minimum hemoglobin level before treatment, previous endometrial curettage, and duration of DNG administration, or uterine or adenomyosis size and the serious bleeding. A DNG-related serious unpredictable bleeding is associated with the structural type of adenomyosis (subtype I) in patients with symptomatic adenomyosis.

## Introduction

Uterine adenomyosis is a common gynecologic disease in premenopausal women and in women of late reproductive age^1^. Adenomyosis is defined as a pathologic condition in which endometrial or endometrium-like structures are present and function in the myometrium. Adenomyosis causes frequent symptoms, including progressive menorrhagia and dysmenorrhea, chronic pelvic pain, dyspareunia, abnormally heavy menstrual bleeding, and interval menstrual bleeding. According to Kishi *et al*., adenomyosis appears to consist of 4 distinct subtypes of different causes: Subtype I adenomyosis (intrinsic), Subtype II adenomyosis (extrinsic), Subtype III adenomyosis (intramural) and Subtype IV adenomyosis (indeterminate)^2^. Subtype I is defined as a deep endometrial invasion into the myometrium and occurs in the uterine inner layer without affecting the outer structures, whereas subtype II is defined as a direct infiltration into the outer shell of the uterus from pelvic endometriosis, without affecting the inner structures. Subtype III is surrounded by intact muscular structures. Subtype IV is a diffuse type that cannot be classified into other subtypes^2^.

The current commonly used treatments for adenomyosis include multiple treatments, such as medication and surgery, and their combinations. At this point, the definitive treatment for adenomyosis is hysterectomy^3^, but it is not the treatment of choice for patients who wish to remain fertile. Many treatment modalities including various nonsurgical and surgical options are now available for the treatment of adenomyosis^4^.

Oral contraceptive pills, high doses of progestin, gonadotropin releasing hormone agonist (GnRH-a), levonorgestrel-releasing intrauterine device (LNGIUD), and danazol are hormonal treatment options for both adenomyosis and endometriosis^1^. Progestin is considered a good choice for long-term treatment of adenomyosis because it causes minimal side effects^5^. The administration of a progestin may not cause a hypoestrogenic state^6^. A new progestin called dienogest (DNG), a 19-nortestosterone derivative, has a bioavailability and strong progestational effect due to its high selectivity to the progesterone receptor^7^. In Japan, DNG 2 mg daily has been used for treatment of symptomatic adenomyosis. DNG has long been used to reduce adenomyosis-related dysmenorrhea. However, some cases could be resistant to DNG.

The most common adverse drug reactions were metrorrhagia, occurring in >90% of women, followed by headache, breast discomfort, hot flashes, and depressed mood, each occurring in <10% of women^8^. The bleeding pattern associated with DNG was frequent, but well tolerated^8^. However, medical treatment option, such as continuous progestin therapy, has risks of excessive hemorrhage from the uterus^5^. Therefore, the use of GnRH-a prior to the continuous progestin therapy in patients with adenomyosis was tested by Khan *et al*.^9^. Some data on adverse effects of DNG, especially on bleeding pattern, have been reported^10,11^. Younger age, anemia before treatment, changes of serum estradiol levels after DNG treatment, and the structural type of adenomyosis appear to be at the risk of uterine bleeding. We aimed to retrospectively analyze the risk factors for a DNG-related serious unpredictable bleeding in women with symptomatic adenomyosis.

## Methods

### General information

This retrospective cohort study was approved by the Ethics Committee of the Nara Medical University, and all methods are performed in accordance with the guidelines and regulations of this journal. All patients signed the informed consent form. Women with symptomatic adenomyosis were transferred from primary clinics to tertiary Nara Medical University Hospital between April 2008 and August 2017.

Eligible patients were aged 30 years or older who desired fertility preservation and did not undergo surgical treatments, such as hysterectomy, who completed prior hormone intervention at least 4 weeks prior to study initiation. Women with adenomyosis diagnosed by MRI were recruited. All eligible patients underwent MRI within one month prior to initiation of treatment with DNG therapy. Patients underwent MRI using T1W and T2W sequences prior to therapy to obtain an accurate evaluation of pelvic disease. MR images were obtained on a 3 T system (Magnetom Verio or Skyra, Siemens Healthcare, Erlangen, Germany). We performed retrospective chart review on 84 patients with adenomyosis diagnosed by MRI who were treated with DNG therapy. Patients’ medical records were anonymous. The therapeutic dose of DNG for the treatment of adenomyosis was 2 mg orally every day.

‘Serious unpredictable bleeding’ was defined as unpredictable, excessive blood loss which interferes with a woman’s physical, social, emotional and/or material quality of life^12^. A DNG-related serious unpredictable bleeding episode fulfilled one of the following criteria: acute overt bleeding associated with a decrease in hemoglobin of at least 2 g per deciliter (g/dl) or a hemoglobin level of 8 g/dl or less if no baseline hemoglobin level was available. Severe dysmenorrhea was defined as dysmenorrhea unresponsive to non-steroidal anti-inflammatory drugs and in bed for 1 day or more.

**Figure 1:**
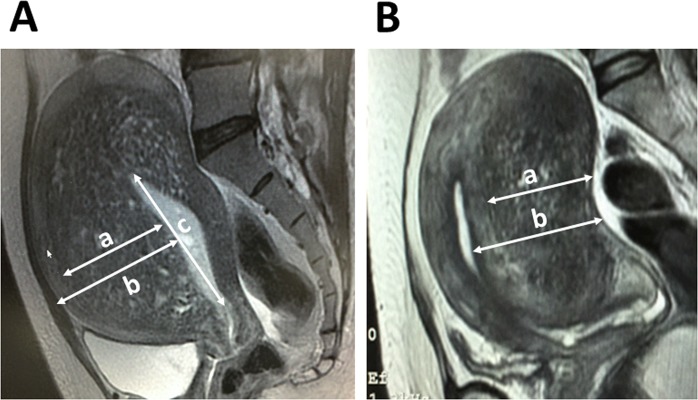
Subtype I and subtype II adenomyosis. T2-weighted magnetic resonance image (sagittal section) of subtype I (intrinsic, (**A**) adenomyosis and subtype II (extrinsic, (**B**) adenomyosis. a, Maximum diameter of adenomyosis-associated lesion with punctate high-intensity myometrial foci (adenomyosis thickness); b, maximum diameter of myometrial wall thickness (myometrial thickness); and c, maximum distance between the uppermost part of the uterine cavity and internal os (cavity longitudinal distance).

The women’s age, body mass index (BMI), parity, severe dysmenorrhea, minimum hemoglobin level before and after therapy, prior therapy, previous cesarean delivery, previous endometrial curettage, presence of endometriotic cyst, presence of leiomyoma, subtype I adenomyosis, maximum diameter of adenomyosis-associated lesion (adenomyosis thickness), maximum distance between the uppermost part of uterine cavity and internal os (cavity longitudinal distance), and maximum diameter of myometrial wall thickness (myometrial thickness) were all recorded^13^ as showed in Table 1. Adenomyosis thickness, cavity longitudinal distance and myometrial thickness were described in Fig. 1.

**Table 1:** Baseline characteristics and univariate analysis of risk factors for dienogest-related serious unpredictable bleeding in patients with symptomatic adenomyosis. IQR, interquartile ranges.

Parameter	The serious bleeding group	The non-serious bleeding group	p value
	(n = 14)	(n = 23)	
Age (years, mean (SD))	40.6 (4.7)	40.8 (4.3)	0.845
BMI (kg/m^2^, mean (SD))	20.7 (3.1)	21.5 (4.4)	0.583
Nulliparous (n (%; 95% CI))	7 (50%)	12 (52%)	1.000
Severe dysmenorrhea	14 (100%)	20 (87%)	0.275
Prior therapy	6 (43%)	10 (43%)	1.000
Previous cesarean delivery	0 (0%)	5 (21.7%)	0.135
Previous endometrial curettage	5 (36%)	11 (48%)	0.471
Presence of hypermenorrhea	8 (57%)	13 (57%)	0.970
Minimum hemoglobin level before DNG treatment (g/dl, mean(SD))	11.2 (1.3)	12.0 (1.2)	0.074
Minimum hemoglobin level after or during DNG treatment (g/dl, mean(SD))	8.3 (1.1)	11.8 (1.2)	0.000
Duration of treatment with DNG (month, IQR, range)	3.5 (IQR, 6.8; range, 2.0–24.0)	15.0 (IQR, 33.0; range, 3.0–96.0)	0.001
The presence of endometriotic cyst	4 (29%)	14 (61%)	0.091
The presence of leiomyoma	4 (29%)	11 (48%)	0.314
Median maximum diameter of adenomyosis associated lesion (mm, IQR, range)	37.5 (IQR, 13.4; range, 22.9–76.3)	33.0 (IQR, 26.4; range, 16.0–72.1)	0.056
Median maximum distance between uppermost part of uterine cavity and internal os (mm, IQR, range)	87.6 (IQR, 40.5; range, 46.0–124.6)	73.0 (IQR, 31.4; range, 55.0–132.3)	0.313
Median maximum diameter of myometrial wall thickness (mm, IQR, range)	45.3 (IQR, 12.9; range, 30.4–76.3)	41.8 (IQR, 32.1; range, 21.0–72.1)	0.090
Subtype I Adenomyosis	8 (57%)	4 (17%)	0.027

### Statistical analysis

SPSS 25.0 (SPSS Inc., Chicago, IL) statistical software was used for the statistical analysis. Data with normal distribution were denoted with mean ± standard deviation, data with skewed distribution were expressed as median value and range, and the count data were presented as the number or percentage. The Mann–Whitney U test was used to compare 2 sets of data with non-normal distribution, and the Fisher’s exact test was used to compare between groups. Cutoff values of the women’s age, BMI, adenomyosis thickness, cavity longitudinal distance, and myometrial thickness were calculated with the highest sensitivity and specificity for predicting the two groups based on the receiver operator characteristic (ROC) curve analysis. Differences with P < 0 .05 were considered statistically significant.

## Results

**Figure 2:**
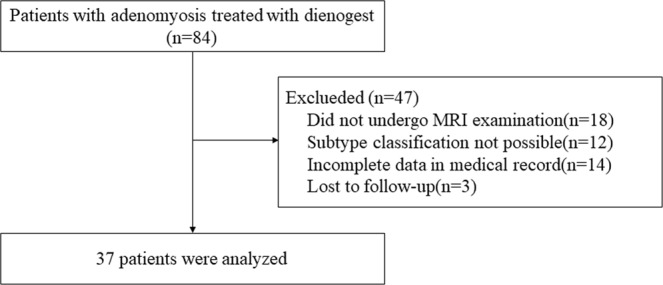
Flow diagram.

Out of the 84 women who were included in this study, 18 patients did not undergo MRI examination before starting DNG therapy. Twelve women with adenomyosis who could not satisfy the subtype I and II criteria were excluded from the analysis. All these 12 cases were subtype IV adenomyosis which may be a heterogeneous mixture of advanced disease. There was no case of subtype III adenomyosis in the patient group. Three women were lost to follow-up. Furthermore, 14 patients lacking hemoglobin levels have been excluded from the analysis. Finally, 37 eligible subjects were included in the analysis (Fig. 2). These patients had an average age of 40 ± 9 years, ranging from 30 to 49. Among them, 6 patients had delivered once, and 11 patients had delivered twice or more. 5 patients had a history of cesarean section at their lower uterine segments. Of these, 3 patients underwent cesarean section once and 2 patients underwent cesarean section twice. 5 patients had a history of endometrial curettage. 16 patients had prior hormonal therapy. Prior to DNG therapy, 16 patients were administered oral contraceptive pills (n = 8) or GnRH-a (n = 7) or pills prior to GnRH-a (n = 1) in the primary clinics. The median duration of treatment with DNG therapy was 11 months. Patients were divided into two groups: the serious bleeding group consisting of patients with serious unpredictable bleeding (n = 14) and the non-serious bleeding group (n = 23). The clinical and imaging characteristics were analyzed and compared between the two groups (Table 1). ROC analysis showed that the optimal cutoff values for women’s age, BMI, cavity longitudinal distance, myometrial thickness, and adenomyosis thickness were 40 years, 20 kg/m^2^, 76 mm, 38.5 mm, and 30.2 mm, respectively, and the areas under their corresponding ROC were 0.432, 0.483, 0.586, 0.614, and 0.646, respectively. Univariate analysis was performed to predict the risk factors of serious unpredictable bleeding. There were no significant differences in women’s age, BMI, parity, dysmenorrhea intensity, prior therapy, previous cesarean delivery, and previous endometrial curettage between the serious bleeding and non-serious bleeding groups. A significant difference was observed with minimum hemoglobin level after or during DNG treatment (8.3 g/dl vs. 11.8 g/dl, P = 0.000) and duration of treatment with DNG (3.5 months vs. 15.0 months, P = 0.001) between the two groups. Patients with subtype I adenomyosis were significantly associated with serious bleeding (P = 0.027). The serious bleeding group was more likely to have increased thickness of the adenomyotic lesions compared to the non-serious bleeding group; however, the difference was not statistically significant. There was no significant difference between the groups with respect to the presence of endometriotic cyst or leiomyoma, uterine cavity longitudinal distance, and myometrial thickness.

## Discussion

In this study, we investigated the risk factors for the DNG-related serious unpredictable bleeding in patients with symptomatic adenomyosis. Serious bleeding occurred in 30.4% of patients treated with continuous DNG therapy during the first 6 months. We found that serious bleeding was associated with the structural type of adenomyosis, suggesting that subtype I (intrinsic) adenomyosis may be a predictor of serious bleeding risk in women receiving DNG therapy.

First, subtype I (intrinsic) adenomyosis was strongly related to the grouping criteria for bleeding. Women with subtype I adenomyosis are more likely to bleed than those with subtype II (extrinsic) adenomyosis when taking continuous DNG. Subtype I adenomyosis has an intimate relationship with inner structural components of the uterus, such as the endometrium and the junctional zone, without affecting the outer structures. In contrast, subtype II adenomyosis develops in the uterine outer layer without affecting the inner structures. Menstruation is triggered by the withdrawal of progesterone, which results in pronounced spiral arteriole constriction in the functional layer of the endometrium, whereas DNG-associated abnormal uterine bleeding occurs from fragile endometrial microvessels^14^. In patients with subtype I adenomyosis, these damaged microvessels are contiguous with decidualized human endometrial stromal cells at the inner myometrium, leading to hemorrhage, and eventually, heavy bleeding. Thus, DNG-induced microvascular damage in subtype I may contribute to excessive genital bleeding. This finding provides a novel mechanistic approach that continuous DNG therapy increases the risk of serious bleeding events in patients with subtype I adenomyosis. This finding was supported by recent data suggesting that DNG remains as an alternative option in the treatment of subtype II adenomyosis^11^.

Second, thickness of the adenomyotic lesions tends to contribute to the increasing incidence of serious bleeding; however, the difference was not statistically significant. Cirpan *et al*. reported that diffuse adenomyotic foci or increased infiltration depth in myometrial tissues in subjects with adenomyosis is associated with severe anemia^15^. Almost all of the patients treated with DNG experienced metrorrhagia that is likely to occur in the first few months of the therapy^8^. The enlargement of the uterus is due to a benign invasion and infiltration of endometrial glands within the underlying myometrium. The severity and frequency of the symptoms may correlate with lesion extent, such as adenomyosis volume and depth of the ectopic endometrium in the myometrium^1^. In our study, however, there was no significant difference in cavity longitudinal distance and myometrial wall thickness between the two groups.

If the severity of symptoms is associated with the size of the lesion, not only adenomyotic lesion thickness but also myometrial thickness and cavity longitudinal distance would be correlated with the risk of serious bleeding. With the limitation of studying only a small number of cases, the result may indicate a discrepancy between these variables.

Third, conservative treatment, including medical, surgical, and interventional options, is still needed in women with symptomatic adenomyosis who desire fertility preservation. Recognized approaches for protecting the QOL of patients are surgery for removing adenomyosis mass and systemic hormonal treatments for terminating dysmenorrhea^4^. If necessary, even surgery such as adenomyomectomy that emphasizes fertility preservation can be applied, but its implementation is lacking in many resource-limited settings^11^. Currently, DNG is commonly used in progestin therapy in Japan. The results of phase III, randomized, double-blind, multicenter, placebo-controlled study revealed that DNG is effective and well tolerated in the treatment for symptomatic adenomyosis^14^. During the treatment period, almost all of the patients treated with DNG experienced multiple uterine bleeding and irregular spotting^8^. However, no patients had severe anemia. The long-term use of 2 mg of DNG orally each day for one year was also well tolerated and effective in patients with symptomatic adenomyosis^5^. Clinically significant drug-related adverse events include metrorrhagia (96.9%) and hot flush (7.7%), which were tolerable in most cases^5^. DNG has certain disadvantages, such as possible bleeding, but has a significant advantage of retaining fertility. Selecting the best treatment for adenomyosis that has the potential of increasing efficacy and decreasing toxicity on an individual basis is required.

Finally, the present study has shortcomings because of the relatively small sample size. Therefore, it is only possible to deduce a hypothesis that the adverse effect (serious bleeding) of DNG therapy mainly depends on the localization (subtype I) of adenomyosis lesions. Future studies with case accumulation may increase the power of the study.

In conclusion, subtype I adenomyosis could predict the risk of serious unpredictable bleeding during continuous DNG therapy. This factor may serve as a filter to carefully identify adenomyosis patients with a high risk of hemorrhage and to select further treatment options that are optimal and individualized for these patients.

### Patient Consent for publication

Informed consent was obtained from all individual participants included in the study.

## Supplementary information

Supplementary Dataset 1

## Data Availability

The datasets generated during and analyzed during the current study are available from the corresponding author on reasonable request.
